# A Dual-Gene Colorimetric LAMP Assay for Genus-Level Detection of *Salmonella* and Specific Identification of the Non-Motile Serovar S. Gallinarum Gallinarum

**DOI:** 10.3390/ijms262412083

**Published:** 2025-12-16

**Authors:** Safae Skenndri, Fatima Ezzahra Lahkak, Taha El Kamli, Zineb Agargar, Imane Abdellaoui Maane, Saâdia Nassik

**Affiliations:** 1 Avian Pathology Unit, Department of Veterinary Pathology and Public Health, Hassan II Institute of Agronomy and Veterinary Medicine, Rabat 6202, Morocco; agar.zineb@gmail.com (Z.A.); s.nassik@yahoo.com (S.N.); 2 Biochemistry, Pharmacology, and Toxicology Unit, Department of Veterinary Biological and Pharmaceutical Sciences, Hassan II Institute of Agronomy and Veterinary Medicine, Rabat 6202, Morocco; f.lahkak@iav.ac.ma (F.E.L.); elkamlit@yahoo.fr (T.E.K.); 3 Independent Researcher, Rabat 12040, Morocco; abdellaoui.iman@gmail.com

**Keywords:** non-motile *Salmonella*, colorimetric isothermal loop amplification, cloacal swabs, point-of-care, field deployable, poultry

## Abstract

*Salmonella* enterica serovar Gallinarum is a non-motile serovar and is the causative agent of fowl typhoid, and poses a major challenge to poultry production, particularly where rapid diagnostics are lacking. Existing methods are either time-consuming or fail to distinguish motile from non-motile serotypes. We developed a dual-target colorimetric LAMP that detects *Salmonella* spp. via *invA* and discriminates S. Gallinarum via *TRX* (a taxon-restricted sequence), using two separate singleplex reactions. Specificity testing confirmed 100% accuracy, with exclusive amplification of S. Gallinarum through *TRX*. Analytical sensitivity was comparable to real-time PCR, detecting down to 2.41 CFU/µL (*invA*) and 1.65 CFU/µL (*TRX*). Applied to cloacal swabs from experimentally infected chickens (n = 12), the assay consistently outperformed bacteriological culture, detecting up to 25% more positives during early infection when bacterial loads were low or cells were non-culturable. This dual-target LAMP provides a rapid, sensitive, and serovar-discriminating diagnostic tool with strong potential for point-of-care use and real-time surveillance in poultry farms, thereby improving sanitary control of fowl typhoid and reducing associated economic losses.

## 1. Introduction

*Salmonella* enterica serovar Gallinarum (S. Gallinarum) is the causative agent of fowl typhoid, a systemic disease that affects poultry and results in high mortality, decreased productivity, and significant economic losses, particularly in low- and middle-income countries where biosecurity measures are often limited [1,2]. As a non-motile serovar adapted to avian hosts, S. Gallinarum poses a unique challenge to control programs due to its capacity for silent transmission and persistent colonization of flocks [3]. Outbreaks of fowl typhoid remain a major concern in poultry production systems, especially where routine screening and rapid intervention measures are lacking. From a regulatory perspective, fowl typhoid is recognized as a notifiable disease by the World Organization for Animal Health (WOAH) and, in Morocco, is legally listed under Law No. 49–99 on the sanitary protection of poultry holdings (Dahir No. 1-02-119 of 13 June 2002), which mandates the declaration of contagious poultry diseases to the competent authorities [4,5,6].

Diagnosis of S. Gallinarum infections traditionally relies on bacteriological isolation, followed by biochemical identification and serotyping based on antigenic formulae. And while considered the gold standard, culture-based methods are labor-intensive, time-consuming (often requiring several days), and dependent on the viability of the organism. Moreover, molecular assays targeting conserved *Salmonella* genes, such as *invA*, offer improved sensitivity and speed, but cannot differentiate between motile and non-motile serotypes [7,8]. This limits their utility in targeted surveillance programs for fowl typhoid, where distinguishing S. Gallinarum from other (motile) *Salmonella* enterica subspecies is crucial for epidemiological and control purposes in poultry flocks.

Given the need for rapid, accurate, and field-adaptable diagnostic tools, point-of-care molecular methods are gaining attention as promising alternatives for *Salmonella* detection (and other prominent poultry diseases like Newcastle Disease, Avian Influenza, Necrotic Enteritis, etc.) in poultry farms [9,10,11]. Loop-mediated isothermal amplification (LAMP) is particularly attractive due to its simplicity, rapid turnaround time, high sensitivity, and isothermal nature, which eliminates the need for thermocycling equipment [12,13]. Several LAMP assays have been developed for *Salmonella* spp. detection [14,15], but most are species-specific and do not distinguish between motile and non-motile serovars. Particularly, there remains a gap in the literature regarding the development of LAMP assays specifically targeting non-motile *Salmonella* serotypes such as S. Gallinarum.

To address this diagnostic gap, we developed a dual-target colorimetric LAMP assay designed to detect *Salmonella* at the genus level using the *invA* gene, and to discriminate S. Gallinarum biovars Gallinarum through amplification of a Taxon-Restricted eXclusive (*TRX*) sequence. Although BLAST (version 2.17.0) analysis indicated that the *TRX* primers also align with S. Pullorum, only S. Gallinarum biovars Gallinarum was included in the present validation due to strain availability. The use of a colorimetric-based detection enables naked-eye visual interpretation without the need for specialized instrumentation, supporting its potential application as a point-of-care screening tool in both laboratory and field settings. This study describes the development, optimization, and evaluation of this dual LAMP assay, including comparisons with real-time PCR and conventional culture methods, as well as its performance on samples collected from experimentally infected birds.

## 2. Results

### 2.1. Specificity of the Assay

**Figure 1:**
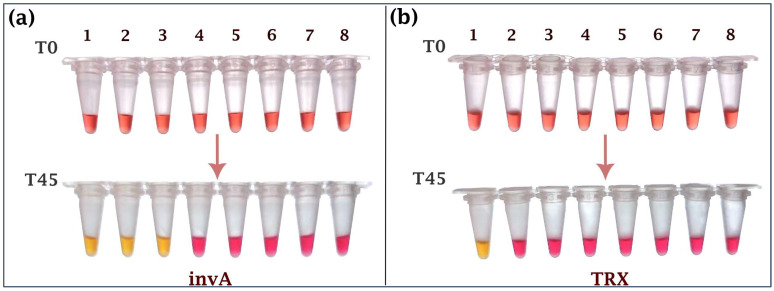
Specificity test for the dual-gene colorimetric LAMP assay, targeting the *Salmonella* spp. specific gene (*invA*) (**a**), and the *Salmonella* Gallinarum discriminatory gene (*TRX*) (**b**). 1: *Salmonella* Gallinarum, 2: *Salmonella* Enteritidis, 3: *Salmonella* Typhimurium, 4: *Escherichia coli*, 5: *Staphylococcus aureus*, 6: *Listeria monocytogenes*, 7: *Bacillus cereus*, 8: *Clostridium perfringens*.

The specificity of the assay was first assessed using a panel of *Salmonella* and non-*Salmonella* bacterial strains, each tested in ten independent replicates. Reactions producing a yellow color were considered positive, while those remaining pink were negative. For the *invA* target, amplification was observed exclusively among *Salmonella* strains, confirming its role as a genus-level marker. In contrast, while designed to detect both S. Gallinarum and S. Pullorum, amplification of the *TRX* target occurred solely in S. Gallinarum biovar Gallinarum, as S. Pullorum strains were not available for experimental validation. No cross-reactivity was detected in other *Salmonella* serovars or non-*Salmonella* species (Figure 1).

While BLASTn analysis of individual primers revealed occasional perfect matches in some motile serovars, these binding sites were shown to be non-functional for LAMP. Manual mapping in representative genomes (e.g., S. Enteritidis, S. Typhimurium) confirmed that primers aligned in orientations and at distances incompatible with the required LAMP geometry. Thermodynamic evaluation further indicated that potential hairpins and dimers were structurally unstable or lacked the critical 3′ complementarity needed to trigger non-specific amplification (see Supplementary Tables S1–S3).

Consistently, no amplification was ever observed in non-template controls across experimental runs, reinforcing that the combined *invA* and *TRX* primer sets are specific and robust for their intended targets.

### 2.2. Sensitivity of the Assay

**Figure 2:**
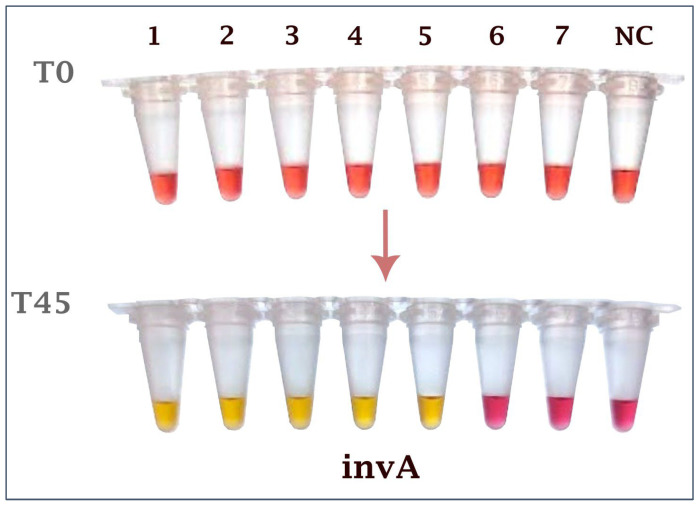
Sensitivity testing for the *invA* gene. The colorimetric LAMP reactions. Ld: 100 kb ladder, 1: 2.41 × 10^4^ CFU/µL, 2: 2.41 × 10^3^ CFU/µL, 3: 2.41 × 10^2^ CFU/µL, 4: 2.41 × 10^1^ CFU/µL, 5: 2.41 × 10^0^ CFU/µL, 6: 2.41 × 10^−1^ CFU/µL, 7: 2.41 × 10^−2^ CFU/µL, NC: Negative control.

**Figure 3:**
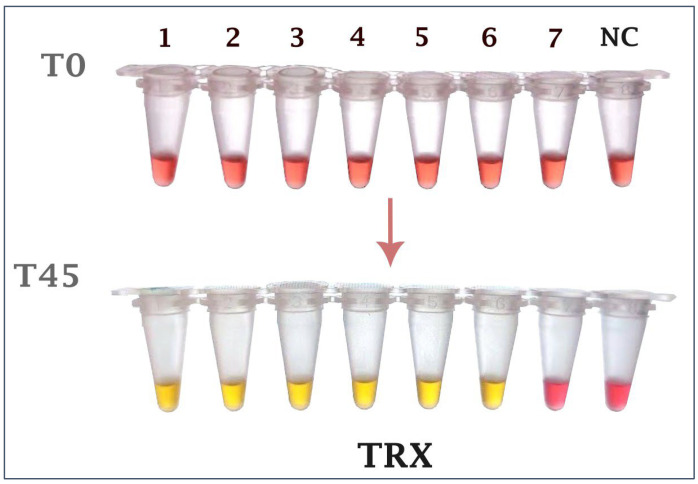
Sensitivity testing for the *TRX* gene. The colorimetric LAMP reactions. Ld: 100 kb ladder, 1: 1.65 × 10^5^ CFU/µL, 2: 1.65 × 10^4^ CFU/µL, 3: 1.65 × 10^3^ CFU/µL, 4: 1.65 × 10^2^ CFU/µL, 5: 1.65 × 10^1^ CFU/µL, 6: 1.65 × 10^0^ CFU/µL, 7: 1.65 × 10^−1^ CFU/µL, NC: Negative control.

To evaluate assay sensitivity, extracted DNA from serial dilutions of overnight culture broths of *Salmonella* Enteritidis and S. Gallinarum were used as templates. At the lowest concentrations tested, each target was run in 20 independent replicates. For the *invA* gene, amplification was consistently obtained at 2.41 CFU/µL in all 20/20 reactions (100%). For the *TRX* gene, amplification was observed at 1.65 CFU/µL in 18/20 reactions (90%). Based on our predefined sensitivity threshold (≥90% positive replicates), these results demonstrate strong analytical sensitivity and reproducibility of the dual-gene assay (Figure 2 and Figure 3). Confirmation of amplification by agarose gel electrophoresis is provided in Supplementary Figures S1 and S2.

### 2.3. Comparison with Real-Time PCR

Given that real-time PCR is a well-established reference method in molecular diagnostics, it was employed as a reference method to evaluate the performance of the colorimetric LAMP assay. The same bacterial DNA templates used for LAMP sensitivity testing were applied in the real-time PCR reactions to ensure consistency. Each dilution was tested in four independent replicates (n = 4), and mean Ct values with standard deviations were calculated.

For the *invA* gene, amplification was consistently observed down to 2.41 × 10^1^ CFU/µL, with a mean Ct of 28.98 ± 0.01, corresponding to an efficiency of 99.2% (R^2^ = 0.999). For the *TRX* gene, amplification was obtained down to 1.65 × 10^1^ CFU/µL, with a mean Ct of 33.88 ± 0.03 and an efficiency of 96.1% (R^2^ = 0.9996). These data confirm excellent reproducibility across replicates.

**Figure 4:**
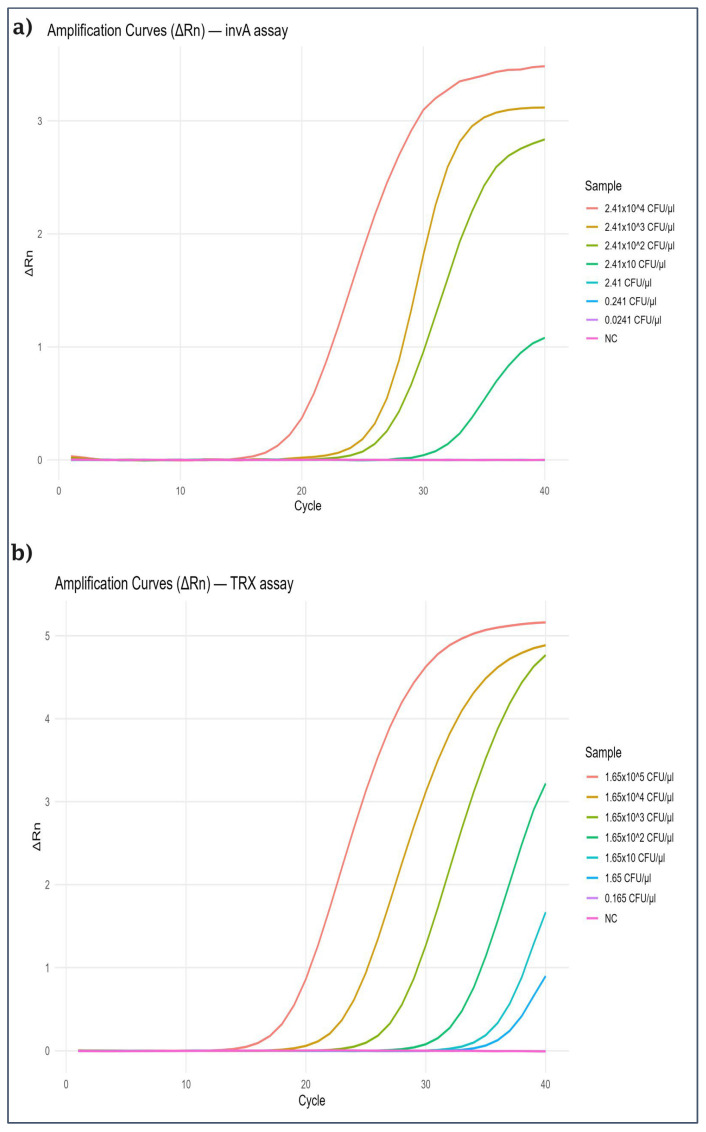
Real-time PCR amplification curves testing the sensitivity of the *invA* gene (**a**) and the *TRX* gene (**b**).

The full set of replicate Ct values, standard deviations, and standard curves for both targets are provided in Supplementary Figure S3 and Table S4. In summary, real-time PCR showed comparable sensitivity to the colorimetric LAMP assay for the *TRX* target, while LAMP displayed slightly improved detection at lower concentrations for the *invA* gene (see Figure 4).

### 2.4. Comparison with Bacteriological Isolation on Cloacal Swabs from Infected Birds

To evaluate the field applicability of the developed assay and assess its potential as a point-of-care diagnostic tool, we tested it on cloacal swabs collected from *Salmonella* Gallinarum-infected chickens. Swabs were collected in duplicate from each bird on alternating days post-infection (1 DPI, 3 DPI, and 5 DPI). In order to simplify the workflow and adapt it to field conditions, DNA was collected using a heat-treatment method instead of a commercial extraction kit. The results obtained with the dual-gene colorimetric LAMP assay were then compared to those of a conventional bacteriological isolation (Table 1). It is important to note that results reported for the colorimetric LAMP assay are representative of both genetic targets; a sample was considered positive only when amplification occurred simultaneously for *invA* and *TRX*.

At 1 DPI, both methods yielded concordant results for 9 birds (6 positives and 3 negatives). Notably, the LAMP assay detected S. Gallinarum in three additional birds (subjects 3, 8, and 9) that were negative by culture. At 3 DPI, agreement was observed in 10 cases (7 positives and 3 negatives), while LAMP identified two further positives (subjects 10 and 12) not detected by bacteriology. By 5 DPI, 9 results were concordant (5 positives and 4 negatives), and the LAMP assay again detected S. Gallinarum in three more individuals (subjects 3, 4, and 11) compared to culture.

## 3. Discussion

The colorimetric LAMP assay developed in this study has proved to be highly specific and sensitive for the detection of *Salmonella* Gallinarum. Amplification of the *invA* gene was observed exclusively in *Salmonella* strains, in accordance with its widely recognized role as a genus-specific marker in recent molecular studies [16]. Furthermore, the *TRX* sequence was amplified solely in S. Gallinarum, with no cross-reactivity detected for other *Salmonella* serovars or non-*Salmonella* species. This highlights the value of the *TRX* sequence as a discriminatory marker for non-mobile *Salmonella* serotypes.

The assay demonstrated high analytical sensitivity, detecting up to 2.41 CFU/µL for *invA* and 1.65 CFU/µL for *TRX*, which are consistent with values reported in recent studies. For instance, the FDA-validated *invA*-based LAMP assay has demonstrated limits of detection ranging from 1.3 to 28 CFU per reaction when tested across different *Salmonella* serovars in pure culture [17]. Another study achieved sensitivities of up to 36 CFU per reaction using LAMP-BART targeting S. Typhimurium in both standard and enriched food matrices.

Wei et al. (2022) reported detection of S. Typhimurium at 7 CFU/mL using a colorimetric LAMP platform, while Regal et al. (2024) achieved an LoD_50_ of 1.8 CFU/25 g in food samples [18,19]. Similarly, another study described a colorimetric assay with an analytical sensitivity of ~3.9 CFU/µL in pure culture, and Balaga et al. (2024) reported ~10-fold higher sensitivity of LAMP compared to PCR [20,21]. The high sensitivity observed here suggests that the assay can reliably detect low bacterial loads, which is of particular importance for the early stages of infection with S. Gallinarum in poultry when shedding may be intermittent or limited [22].

Published *Salmonella* LAMP assays often incorporate a short enrichment step prior to amplification, which is highly effective for boosting sensitivity in complex food or environmental matrices. In contrast, the present assay was evaluated under direct detection conditions, without pre-enrichment, thereby providing an immediate measure of analytical sensitivity. While this difference makes direct numerical comparisons challenging, both approaches are complementary: enrichment-based workflows maximize detection in heavily contaminated or inhibitor-rich samples, whereas direct workflows, such as the one described here, prioritize speed and operational simplicity for point-of-care use. Within these differing contexts, the detection limits obtained here remain consistent in order of magnitude with those reported across *Salmonella* LAMP assays.

When confronted with real-time PCR, the colorimetric LAMP assay displayed comparable performance for the *TRX* gene and even higher sensitivity for the *invA* target. These findings are consistent with recent comparative analyses showing that LAMP can equal or outperform qPCR in terms of detection limits, while demanding less equipment and delivering faster results [23,24]. This reinforces the practical utility of LAMP as a diagnostic tool, particularly in resource-limited veterinary contexts where access to qPCR platforms is often constrained.

A range of molecular assays aimed at distinguishing S. Gallinarum (and the closely related S. Pullorum) have been reported. One-step PCR assays targeting the flhB gene utilized a region deletion unique to these biovars, enabling clear differentiation from other serovars [25]. Another multiplex PCR approach used the following genes: stn, I137_08605 and ratA, with I137_08605 present only in S. Pullorum and S. Gallinarum, and a deletion in ratA distinguishing S. Pullorum, which allowed for serovar identification [26]. Most notably, a FRET-PCR assay directed at the pegB gene, exclusive to S. Pullorum and S. Gallinarum, allowed high-resolution melting curve differentiation [27]. To the best of our knowledge, no LAMP-based assay has previously been developed to differentiate non-motile *Salmonella* serotypes from their motile counterparts. In this context, our *invA*\*TRX*-based colorimetric LAMP assay distinguishes itself with both its specificity and its operational simplicity. By delivering rapid, serovar-specific detection without the need for gel electrophoresis or advanced thermal cycling, it is a strong contender among contemporary molecular tools for field screening and diagnostic of fowl typhoid.

Application of the assay to cloacal swabs from experimentally infected chickens further highlighted its diagnostic potential, as the colorimetric LAMP assay consistently outperformed bacteriological isolation across all sampling days. At 1 DPI, LAMP detected S. Gallinarum in 75% of birds compared with 50% by culture, representing a 25% increase in detection. Similarly, at 3 DPI, detection improved from 58.3% with culture to 75% with LAMP, a gain of 16.7%. By 5 DPI, LAMP identified 66.6% of positives versus 41.6% with culture, again reflecting a 25% improvement. This difference in results between the two approaches reflects the capacity of LAMP to amplify DNA from very low bacterial loads as well as from cells that may be stressed or non-culturable, which often evade detection on selective agar [28]. Comparable observations have been reported in other veterinary and food safety studies, where LAMP and related molecular assays consistently surpassed culture-based methods in sensitivity and early detection capability [23,25].

While the assay performed well under controlled conditions, field matrices can introduce amplification inhibitors (bile salts, humic substances, proteins, polysaccharides) that reduce efficiency and can increase false-negative risk at low loads. Recent work systematically quantified LAMP inhibition by common matrix components, highlighting bile salts, hematin, and humic acid as impactful inhibitors. Simple pre-treatment approaches and matrix-adapted workflows have been shown to mitigate these effects in poultry production environments [29,30].

The colorimetric pH readout used here (phenol-red shift as amplification acidifies the mix) enables instrument-free interpretation, which is well documented for LAMP and widely used in fieldable formats. Moreover, lyophilization of LAMP reagents has been shown to preserve performance after ambient/elevated-temperature storage, supporting cold-chain-independent deployment. Combined with short run-times (<1 h) and minimal equipment, these features suit low-resource and on-farm screening [31,32,33]. And considering that *Salmonella* Gallinarum (fowl typhoid) is recognized by WOAH and appears on official notifiable/reportable disease lists, rapid on-site testing can facilitate timely reporting and control measures in poultry systems, aligning with One Health surveillance goals.

Like other LAMP-based systems, the assay may be susceptible to unintended amplification events, such as non-specific priming or primer-dimer formation, which can generate false-positive signals under suboptimal conditions. This risk was mitigated by rigorous in silico analysis of primer sets, empirical testing of non-template controls, and the inclusion of dual-target confirmation, but it remains a known limitation of LAMP methodologies. Another limitation is the lack of true multiplexing: although the assay simultaneously targets *invA* and *TRX*, these are amplified in separate reactions. Future work could explore the development of multiplex LAMP chemistries or microfluidic formats capable of simultaneous multi-target amplification in a single reaction chamber, further increasing throughput and robustness [34,35]. Alternatively, Lateral Flow Dipstick formats, by labeling primers with distinct tags (e.g., biotin/FAM vs. digoxigenin/biotin) and reading on multiplexed strips, would permit single-reaction detection and serovar discrimination [36]. This work provides a basis for developing a LAMP-LFA test.

The present study introduces several technical advancements. First, it is, to our knowledge, the first report to exploit the *TRX* locus as a diagnostic marker for the specific identification of S. Gallinarum. Second, by combining genus-level detection (*invA*) with a serovar-specific discriminatory target (*TRX*), the assay delivers dual-target confirmation, which enhances specificity and reliability. Third, the use of a colorimetric readout provides instrument-free detection that is particularly suited for resource-limited or field environments, distinguishing this approach from fluorescence- or turbidimetry-based LAMP platforms. Finally, the assay was validated using cloacal swabs from experimentally infected chickens, demonstrating applicability beyond spiked samples and supporting its relevance for real-world diagnostic and surveillance needs. Taken together, these features underscore the novelty of the assay and its potential contribution to poultry health management and food safety within a One Health perspective.

## 4. Materials and Methods

### 4.1. Salmonella gallinarum Challenge and Cloacal Swab Collection

**Figure 5:**
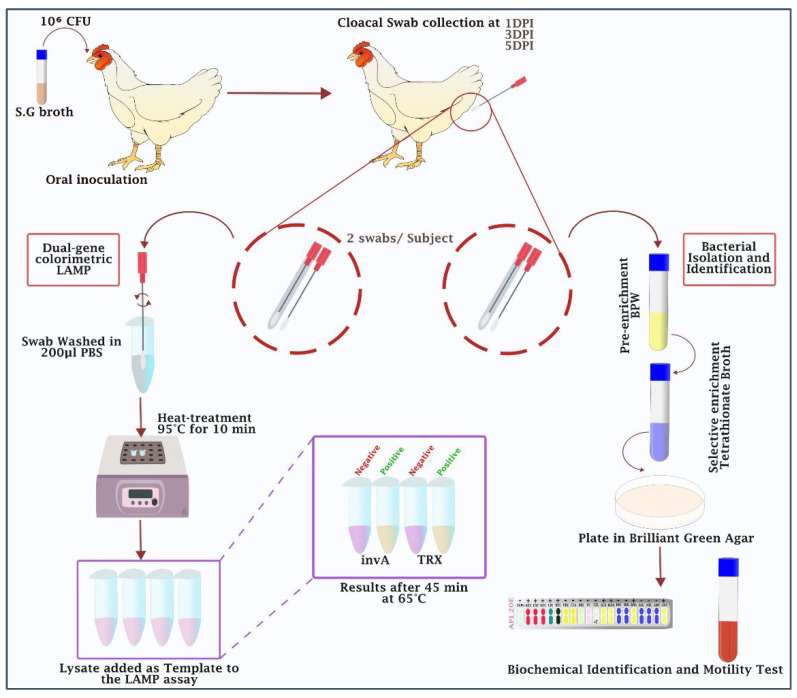
Schematic workflow for the detection of S. Gallinarum via the dual-gene colorimetric LAMP assay and bacterial isolation and identification.

The performance of the colorimetric LAMP assay for detecting *Salmonella* Gallinarum was evaluated in parallel with the standard bacteriological isolation method. A schematic illustration of the workflow is shown in Figure 5.

A total of 24 six-week-old healthy Ross 208 broiler chickens (*Gallus gallus* Domesticus) (all males) were used in this study. Birds were divided into two groups: an infected group (n = 12) and a non-infected control group (n = 12). The experimental unit was an individual bird. Animals were housed in a closed room measuring 2.0 × 2.5 m, with hay litter, and provided feed and water ad libitum.

The infection challenge consisted of oral inoculation with 1 mL of an overnight Buffered Peptone Water culture containing 10^6^ CFU of a field isolate of *Salmonella* enterica serovar Gallinarum. Control birds were mock-inoculated with sterile Buffered Peptone Water.

Cloacal swabs were collected from each bird at 1, 3, and 5 days post-infection (DPI). During sampling, one handler restrained the bird to minimize stress, while a second handler collected two sterile swabs per bird. Samples were immediately processed for DNA collection and bacterial culture isolation.

Birds were observed daily for general health and clinical signs, and no humane endpoints were reached during the study. All experimental procedures were conducted in accordance with the ARRIVE guidelines. Ethical approval was obtained from the Ethics Committee on Animal Science, Animal Health and Veterinary Public Health (CESASPV) of IAV Hassan II (approval number: CESASPV_2025_A13).

### 4.2. Bacteriological Isolation of Salmonella gallinarum from Cloacal Swabs

A modified version of the ISO method for *Salmonella* isolation was applied to adapt it to the specific requirements of S. Gallinarum [37]. As in the ISO protocol, swabs were first pre-enriched in Buffered Peptone Water (Sigma-Aldrich, Poole, UK) for 18 h at 37 °C. Selective enrichment was then performed in Mueller–Kauffmann Tetrathionate Broth Base without novobiocin (Himedia, Mumbai, India) for 20 h at 37 °C, instead of the Rappaport–Vassiliadis and MKTTn broths recommended by ISO, since both novobiocin and high osmolarity can inhibit recovery of S. Gallinarum, particularly at low bacterial loads. Plates were streaked onto Brilliant Green Agar (Biokar, Beauvais, France) rather than XLD agar, as S. Gallinarum is poorly recovered on some standard selective media, and incubated for 24 h. Presumptive colonies were re-plated onto TSA agar and incubated for 18–20 h to ensure purity, then subjected to biochemical confirmation with the API 20E system (Biomérieux, Marcy l’Etoile, France). Finally, motility was assessed using Mannitol Motility Nitrate Medium (Himedia, Mumbai, India) to confirm the non-motile phenotype characteristic of S. Gallinarum.

### 4.3. DNA Collection

#### 4.3.1. Heat-Treatment

For each time point, one swab was processed using conventional bacteriological culture for the isolation and identification of S. Gallinarum (see Section 4.2). The second swab was suspended in 200 µL of PBS (Thermo Scientific, Franklin, MA, USA), and the resulting suspension was subjected to a heat treatment at 95 °C for 10 min using a dry heat block. The lysates were then used directly as templates in the colorimetric LAMP assay.

#### 4.3.2. DNA Extraction

For sensitivity testing, DNA was extracted from bacterial cultures using the PureLink™ Genomic DNA Mini Kit (Invitrogen, Carlsbad, CA, USA).

### 4.4. Colorimetric Buffer Preparation

The colorimetric buffer used for the cLAMP reaction was prepared from ammonium sulfate ((NH_4_)_2_SO_4_, 132.14 g/mol, Merck Millipore, Billerica, MA, USA), magnesium sulfate (MgSO_4_·7H_2_O, 246.47 g/mol, Sigma-Aldrich, Poole, UK), potassium chloride (KCl, 74.55 g/mol, Merck Millipore, Billerica, MA, USA), phenol red (354.38 g/mol, Merck Millipore, Billerica, MA, USA), Tris-HCl (157.56 g/mol, Invitrogen, Carlsbad, CA, USA), and potassium hydroxide (KOH, 56.11 g/mol, Oxford Lab Fine Chem LLP, Maharashtra, India).

Phenol red was included in the reaction buffer as a pH-sensitive indicator. During amplification, the release of pyrophosphate ions leads to proton accumulation and a gradual decrease in pH, shifting the indicator from pink (alkaline/no amplification) to yellow (acidic/positive amplification). This colorimetric change allows direct visual interpretation without the need for instrumentation.

A 2× buffer stock was prepared containing all components at the concentrations shown in Table 2 under “Initial Aliquot Concentration [×2] (mM)”, and the pH was adjusted to 9.0 with KOH. For each 25 µL LAMP reaction, 12.5 µL of the 2× buffer was added to obtain the final 1× concentrations listed under “Final Concentration in Reaction [×1] (mM)”.

### 4.5. Primer Design

The assay employed two genes for detecting *Salmonella* Gallinarum. First, the *invA* gene was used to confirm the presence of *Salmonella* species. *invA*, a well-characterized *invA*sion gene located within the *Salmonella* pathogenicity island 1 (SPI-1), was selected as a non-discriminatory target for general *Salmonella* detection, as it is widely conserved among motile and non-motile serovars and frequently used in molecular assays.

A gene annotated as SPUL_RS24225 in the *Salmonella* enterica subsp. enterica serovar Gallinarum/Pullorum strain RKS5078 (RefSeq: NC_016831.1), predicted to encode a hypothetical protein, was selected as the discriminatory marker. The locus sequence was queried by BLASTn against the NCBI Nucleotide collection (nr/nt) databases. This query confirmed the locus was consistently present in S. Gallinarum and S. Pullorum. For clarity, this locus is referred to throughout as the Taxon-Restricted eXclusive element (*TRX*).

Primers were designed using the PrimerExplorer v5 tool (Eiken Chemical Co., Tokyo, Japan) and synthesized by Eurogentec (Ougrée, Seraing, Belgium). The primer sets for each gene are listed in Table 3.

In silico validation of primers was performed in several steps. Each primer was screened individually using BLASTn against the nucleotide database, and inner-site primer pairs (F2/B2, F1c/B1c, F2/B1c, F1c/B2) were evaluated with Primer-BLAST to identify potential amplicons. For predicted hits, primer positions and orientations were extracted from genome coordinates to assess compatibility with LAMP amplification geometry.

Thermodynamic stability of primers was evaluated using OligoAnalyzer (Integrated DNA Technologies, IDT) under reaction-like ionic conditions (50 mM Na^+^, 10 mM Mg^2+^). For each primer, hairpin ΔG, self-dimer ΔG, and melting temperatures (Tm) were recorded. Cross-dimer interactions were also analyzed, with particular attention to 3′–3′ complementarity.

Finally, primer sets were experimentally validated by monitoring non-template controls (NTCs) across more than 50 independent assay runs.

### 4.6. Colorimetric LAMP Reaction Composition

This assay consists of two separate LAMP reactions per sample to prevent primer-dimer interference. Each reaction targets a different gene: one contains primers specific for the *invA* gene, and the other for the *TRX* sequence. The LAMP reaction mixture (25 µL total volume) includes 12.5 µL of colorimetric buffer, 1 µL of the BstY Polymerase (8 U/µL), and 3.5 µL of dNTPs (1.6 mM each) (NZYTech, Lisbon, Portugal), 2.5 µL of primer mix (final concentrations: 0.2 µM F3/B3, 1.6 µM FIP/BIP, and 0.4 µM LF/LB), 0.5 µL of nuclease-free water, and 5 µL of the sample. Amplification was carried out at 65 °C for 45 min using a PTC-100 programmable thermal controller (MJ Research Inc., Waltham, MA, USA). Results are deemed positive when a yellow color is displayed, and negative when a pink color appears.

### 4.7. The Assay’s Specificity

The colorimetric LAMP assay’s specificity was tested for both genes, *invA* and *TRX*, against *Salmonella* and non-*Salmonella* bacteria (for *invA*), and motile and non-motile serotypes (for *TRX*). Each strain was tested in ten independent replicates. The bacterial species and serovars used were: *Salmonella* Enteritidis, *Salmonella* Typhimurium, *Salmonella* Gallinarum biovar Gallinarum (local strain), *Escherichia coli*, *Clostridium perfringens*, *Staphylococcus aureus*, *Listeria monocytogenes*, and *Bacillus cereus*. All strains were obtained from the culture collection of the Avian Pathology Unit, IAV Hassan II.

### 4.8. The Assay’s Sensitivity

The sensitivity of the assay was evaluated using extracted DNA from cultures of *Salmonella* Enteritidis and a local strain of *Salmonella* Gallinarum. Serial dilutions of overnight culture broths were prepared, and the lowest detectable concentration for each target gene was tested in 20 independent replicates to assess reproducibility. For this study, the sensitivity threshold was defined as the lowest concentration at which ≥90% of replicates yielded positive amplification.

### 4.9. Confirmation of Amplification in LAMP Reactions

To confirm that amplification occurred in the LAMP reaction that displayed a yellow color, a gel electrophoresis was run. A 1.6% gel was prepared using a molecular-grade agar (Thermo Scientific™, Waltham, MA, USA). Each well contained 5 µL of LAMP products, 2 µL of Blue Juice (Invitrogen™, Carlsbad, CA, USA), and 3 µL of nuclease-free water. A 100 bp Ladder was used as a molecular weight marker (Invitrogen™, Waltham, MA, USA). The electrophoresis was run at 80 V for 45 min.

### 4.10. Real-Time PCR

Real-time PCR was performed to evaluate the analytical sensitivity of the colorimetric LAMP assay in comparison to a standard molecular detection method. Sensitivity was assessed separately for each target gene (*invA* and *TRX*). Each 20 µL reaction contained 10 µL of SsoFast EvaGreen^®^ Supermix (Bio-Rad, Hercules, CA, USA), 0.4 µM of each forward and reverse primer, 7 µL of nuclease-free water, and 5 µL of DNA extracted from *Salmonella* cultures. Primer sequences used were as follows: *invA* (F: 5′-GGATTGGACCTCAAGTGTA-3′; R: 5′-GTCCCGGCTTTATGAACG-3′) and *TRX* (F: 5′-ACGCGTTCTGAACCTTTGG-3′; R: 5′-CGTTTCCTGCGGTACTGTT-3′). Amplification was carried out on a QuantStudio™ 5 Real-Time PCR System (Thermo Fisher Scientific™, Waltham, MA, USA) using the following cycling conditions: initial denaturation at 95 °C for 10 s, followed by 40 cycles of 95 °C for 30 s, gene-specific annealing (56 °C for *invA* and 59 °C for *TRX*) for 30 s, and extension at 72 °C for 30 s.

Each dilution point was tested in four independent replicates (n = 4), and Ct values were recorded to calculate mean values and standard deviations. Standard curves were generated by plotting log_10_ concentration versus mean Ct, and PCR efficiency was determined from the slope of the regression line.

## Figures and Tables

**Table 1:** Comparison of the dual-gene colorimetric LAMP assay (L) and bacteriological culture (C) for the detection of *Salmonella* Gallinarum in cloacal swabs collected at 1, 3, and 5 days post-infection in experimentally infected chickens.

Bird		1		2		3		4		5		6		7		8		9		10		11		12	
Method		C	L	C	L	C	L	C	L	C	L	C	L	C	L	C	L	C	L	C	L	C	L	C	L
DPI	1	+		+		−		+		+		−		+		−		−		−		−		+	
	3	+		+		−		+		+		+		+		−		+		−		−		−	
	5	+		−		−		−		+		−		+		−		−		+		−		+	

L: dual gene colorimetric LAMP results [Yellow: Positive for both genes; Pink: Negative]. C: Culture results [(+) indicate a positive culture and identification of S. Gallinarum. (−) indicate a negative culture for S. Gallinarum].

**Table 2:** Composition of the colorimetric lamp buffer.

Component	Initial Aliquot Concentration [×2] (mM)	Final Concentration in the Reaction [×1] (mM)
(NH_4_)_2_SO_4_	20	10
MgSO_4_	20	10
KCl	60	30
Phenol red	0.4	0.2
Tris-HCl	0.8	0.4

**Table 3:** Primer sequences for the *invA* and *TRX* genes.

Gene	Primer	Sequence	Length
*invA* (NC_011294.1)	F3	5′-ACGCGTTCTGAACCTTTGG-3′	19
	B3	5′-CGTTTCCTGCGGTACTGTT-3′	19
	FIP	5′-GCCACGTTCGGGCAATTCGTTATAAACTGGACCACGGTGACA-3′	42
	BIP	5′-AATTTCACCGGCATCGGCTTCACGCTCTTTCGTCTGGCATTA-3′	42
	LF	5′-CGGTGGGTTTTGTTGTCTTCTCTA-3′	24
	LB	5′-TCAAGATAAGACGGCTGGTACTGAT-3′	25
*TRX* (SPUL_RS24225, NC_016831.1)	F3	5′-GGATTGGACCTCAAGTGTA-3′	19
	B3	5′-GTCCCGGCTTTATGAACG-3′	18
	FIP	5′-GTGGGTACTTTGCCGGATGGGGTCTACCATCAGAACTGC-3′	39
	BIP	5′-CGTCCCGTAACATAATTATTGTCGATGATGAGGCTAACAAGGATT-3′	45
	LF	5′-GCACAGTGATTGTGCGTGATG-3′	21
	LB	5′-CCTTAACATCGCTAGGGGATAAGTT-3′	25

## Data Availability

The original contributions presented in this study are included in the article/Supplementary Materials. Further inquiries can be directed to the corresponding author.

## Supplementary Materials

The following supporting information can be downloaded at: https://www.mdpi.com/article/10.3390/ijms262412083/s1.

## Abbreviations

The following abbreviations are used in this manuscript:

S. Gallinarum	*Salmonella* enterica serovar Gallinarum
LAMP	Loop-mediated isothermal amplification
*invA*	A conserved *Salmonella invA*sion gene in SPI-1 (genus-specific marker)
*TRX*	Taxon-Restricted eXclusive sequence (serovar-specific marker for S. Gallinarum)
CFUs	Colony-Forming Units (measure of viable bacteria)
PCR	Polymerase Chain Reaction
qPCR/real-time PCR	Quantitative real-time PCR
DPI	Days Post-Infection
NC	Negative Control
Ld	Ladder (molecular weight size marker in gel electrophoresis)
API 20E	Analytical Profile Index 20E system (biochemical ID kit for Enterobacteriaceae)
CESASPV	Comité d’Éthique en Sciences et Santé Animales et Santé Publique Vétérinaire (IAV Hassan II)
IAV Hassan II	Institut Agronomique et Vétérinaire Hassan II (Morocco)
ISO 6579-1:2017	International Organization for Standardization method for *Salmonella* detection
PBS	Phosphate-Buffered Saline
ARRIVE guidelines	Animal Research: Reporting of In Vivo Experiments
SPI-1	*Salmonella* Pathogenicity Island-1
F3/B3/FIP/BIP/LF/LB	LAMP primer types (Forward/Backward outer, Forward/Backward Inner Primer, Loop Forward/Backward)
BstY Polymerase	DNA polymerase enzyme used in isothermal amplification
Tris-HCl	Tris(hydroxymethyl)aminomethane hydrochloride buffer
MgSO_4_	Magnesium sulfate
KCl	Potassium chloride
KOH	Potassium hydroxide
(NH_4_)_2_SO_4_	Ammonium sulfate
FRET-PCR	Fluorescence Resonance Energy Transfer PCR
