# Supplementary material for: A Dual-Gene Colorimetric LAMP Assay for Genus-Level Detection of Salmonella and Specific Identification of the Non-Motile Serovar S. Gallinarum Gallinarum
Source: Int J Mol Sci. 2025 Dec 16;26(24):12083. doi: 10.3390/ijms262412083 (PMC12732879; doi:10.3390/ijms262412083)
Supplement: Supplementary file 1 [file ijms-26-12083-s001.zip › ijms-3871692-supplementary.pdf]

A Dual-Gene Colorimetric LAMP Assay for Genus-Level Detection of *Salmonella* and Specific Identification of the Non-Motile Serovar *S. gallinarum* Gallinarum

Safae Skenndri [1,*], Fatima Ezzahra Lahkak [2], Taha El Kamli [2], Zineb Agargar [1], Imane Abdellaoui Maane [3,⁂] and Saâdia Nassik [1,⁂]

[1]Avian Pathology Unit, Department of Veterinary Pathology and Public Health, Hassan II Institute of Agronomy and Veterinary Medicine, Rabat 6202, Morocco; safaesk7@gmail.com (S.S.); agar.zineb@gmail.com (Z.A.); s.nassik@yahoo.com (S.N.)

[2]Biochemistry, pharmacology, and toxicology unit, Department of Veterinary Biological and Pharmaceutical Sciences, Hassan II Institute of Agronomy and Veterinary Medicine, Rabat 6202, Morocco; f.lahkak@iav.ac.ma (F.E.L.); elkamlit@yahoo.fr (T.E.K.)

[3]Independent Researcher, Rabat, Morocco; abdellaoui.iman@gmail.com

*Correspondence: safaesk7@gmail.com; s.skenndri@iav.ac.ma

⁂ These authors contributed equally to this work.

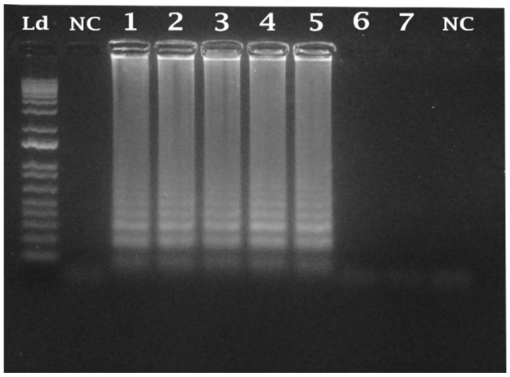

Figure S1. Sensitivity testing for the invA gene. Confirmation of amplification with an electrophoresis gel. Ld: 100 kb ladder, 1: 2.41×10^{4} CFU/µl, 2: $2.41\text{x}10^{3}$ CFU/µl, 3: $2.41\text{x}10^{2}$ CFU/µl, 4: $2.41\text{x}10^{1}$ CFU/µl, 5: $2.41\text{x}10^{0}$ CFU/µl, 6: $2.41\text{x}10^{-1}$ CFU/µl, 7: $2.41\text{x}10^{-2}$ CFU/µl, NC: Negative control.

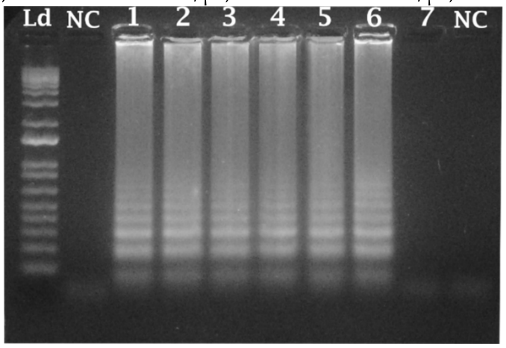

Figure S2. Sensitivity testing for the TRX gene. Confirmation of amplification with an electrophoresis gel. Ld: 100 kb ladder, 1: $1.65\text{x}10^{5}$ CFU/µl, 2: $1.65\text{x}10^{4}$ CFU/µl, 3: $1.65\text{x}10^{3}$ CFU/µl, 4: $1.65\text{x}10^{2}$ CFU/µl, 5: $1.65\text{x}10^{1}$ CFU/µl, 6: $1.65\text{x}10^{0}$ CFU/µl, 7: $1.65\text{x}10^{-1}$ CFU/µl, NC: Negative control.

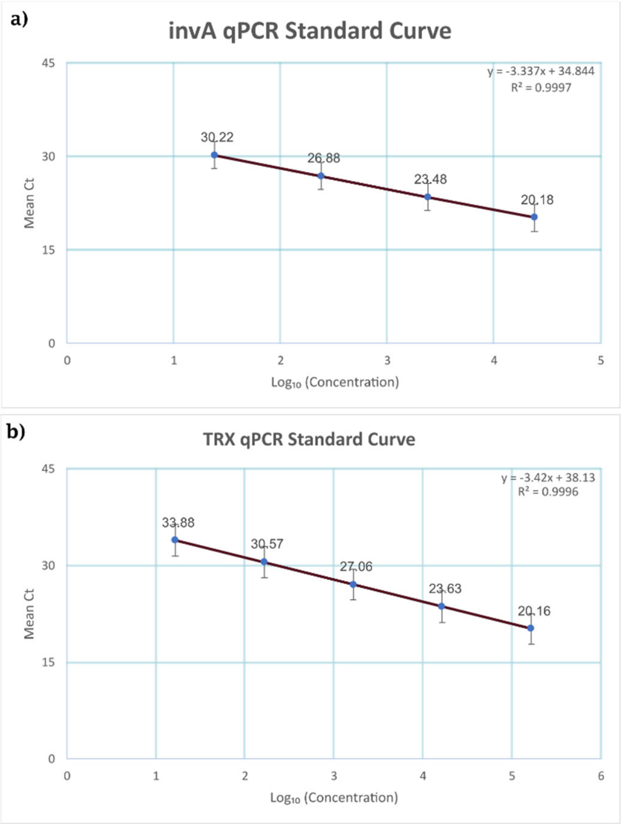

Figure S3. Standard curves for real-time PCR amplification of Salmonella target genes. (a) invA and (b) TRX. Each data point represents the mean cycle threshold (Ct) value from four independent replicates (n = 4), plotted against the $\log_{10}$-transformed bacterial concentration (CFU/µl). Error bars indicate standard deviations. Regression equations, coefficients of determination (R^2) are shown on each panel

Table S1. In-silico analysis of TRX and invA primers

Gene	Primer name	Sequence (5′–3′)	Top BLAST hit (organism)	Identity (%)	Coverage (%)	Predicted amplicons outside target	Hairpin ΔG (kcal/mol)	Hairpin Tm	Self-Dimer ΔG (kcal/mol)	Notes
invA	**F3**	ACGCGTTCTGAACCTTTGG	Salmonella spp.	100%	100%	none	0.45	14.7	-13.05	Acceptable, Slightly strong dimer potential, no 3′ overlap
	**F2**	ATAAACTGGACCACGGTGACA	Salmonella spp.	100%	100%	none	-0.41	32.9	-5.02	Acceptable
	**F1c**	GCCACGTTCGGGCAATTCGTT	Salmonella spp.	100%	100%	none	-1.17	44.2	-6.3	Acceptable
	**LF**	CGGTGGGTTTTGTTGTCTTCTCTA	Salmonella spp.	100%	100%	none	2.71	-45.1	-3.61	Acceptable
	**B3**	CGTTTCCTGCGGTACTGTT	Salmonella spp.	100%	100%	none	0.28	22.3	-3.65	Acceptable
	**B2**	CGCTCTTTCGTCTGGCATTA	Salmonella spp.	100%	100%	none	0.2	21.8	-3.61	Acceptable
	**B1c**	AATTTCACCGGCATCGGCTTCA	Salmonella spp.	100%	100%	none	-3.2	62.3	-9.75	Acceptable, Slightly strong dimer potential, no 3′ overlap
	**LB**	TCAAGATAAGACGGCTGGTACTGAT	Salmonella spp.	100%	100%	none	0.25	17.9	-3.65	Acceptable

	**FIP**	GCCACGTTCGGGCAATTCGTTATAAACTGGACCACGGTGACA	Salmonella spp.	100%	100%	none	-1.73	39.9	-7.29	Acceptable
	**BIP**	AATTTCACCGGCATCGGCTTCACGCTCTTTCGTCTGGCATTA	Salmonella spp.	100%	100%	none	-2.26	37.3	-9.75	Acceptable 3' complementarity is minimal
**TRX**	**F3**	GGATTGGACCTCAAGTGTA	S. Gallinarum and S. Pullorum	100%	100%	none	-1.04	38	-3.9	Acceptable
	**F2**	GGTCTACCATCAGAACTGC	S. Gallinarum and S. Pullorum	100%	100%	none	-1.05	33.3	-4.41	
	**F1c**	GTGGGTACTTTGCCGGATGG	S. Gallinarum and S. Pullorum	100%	100%	none	-0.72	35.1	-9.75	Acceptable considering FIP primer complexity
	**LF**	GCACAGTGATTGTGCGTGATG	S. Gallinarum and S. Pullorum	100%	100%	none	-4.76	63.9	-8.39	Acceptable

	**B3**	GTCCCGGCTTTATGAACG	S. Gallinarum and S. Pullorum	100%	100%	none	0.01	24.8	-9.75	Acceptable
	**B2**	TGATGAGGCTAACAAGGATT	S. Gallinarum and S. Pullorum	100%	100%	none	1.37	-3.9	-3.14	Acceptable
	**B1c**	CGTCCCGTAACATAATTATTGTCGA	S. Gallinarum and S. Pullorum	100%	100%	none	0.27	21.2	-10.23	Acceptable 3' complementarity is minimal (no 3′ overlap)
	**LB**	CCTTAACATCGCTAGGGGATAAGTT	S. Gallinarum and S. Pullorum	100%	100%	none	0.05	24.1	-4.85	Acceptable
	**FIP**	GTGGGTACTTTGCCGGATGGGGTCTACCATCAGAACTGC	S. Gallinarum and S. Pullorum	100%	100%	none	-5.58	53.3	-9.75	Acceptable. 3' complementarity is minimal. Internal

										complementarity only
	**BIP**	CGTCCCGTAACATAATTATTGTCGATGATGAGGCTAACAAGGATT	S. Gallinarum and S. Pullorum	100%	100%	none	-1.01	36.8	-10.23	Acceptable. 3' complementarity is minimal. Internal complementarity only

In-silico analysis of TRX and invA primers. Each primer was queried against the nt database (BLASTn) to determine top hits, coverage, and identity. Primer-BLAST was used to predict possible amplicons, including mixed inner-site pairs. Thermodynamic stability was evaluated using OligoAnalyzer (IDT), reporting hairpin ΔG, hairpin Tm, self-dimer ΔG, and predicted cross-dimers between primer combinations. Cases with $\Delta G \leqslant -9$ kcal/mol are highlighted, with annotation of 3′ overlap presence or absence. No working complete sets of primers that could be used in the LAMP reaction were found in motile serovars, which is consistent with the experimental results demonstrating the assay's specificity.

Table S2. Primer-BLAST predicted amplicons for TRX and invA primer pairs

Primer pair tested	Target genome(s) with predicted amplicon	Amplicon size (bp)	Non-target genomes with predicted amplicon	Notes
**TRX F2/B2**	*S. Gallinarum*<br>S. Pullorum<br>Accesion number:<br>CP068386.1<br>CP003786.1<br>CP003047.1<br>CP019035.1<br>CP116616.1	141	*S. Berta*<br>*S. Enteritidis*<br>*S. Typhimurium*<br>*Accession number:*<br>*CP045956.1*<br>*CP045955.1*<br>*CP088136.1*<br>*CP082714.1*	Prediction based on a simplified PCR model. Full LAMP primer geometry (orientation/spacing) is incorrect in non-target genomes, preventing amplification.

	CP100648.1<br>CP118130.1<br>CP118128.1<br>CP118126.1<br>CP118124.1<br>CP118122.1<br>CP118132.1<br>CP118120.1<br>CP118118.1<br>CP118116.1<br>CP118114.1<br>CP118112.1<br>CP077760.1<br>HQ014666.1<br>HQ014667.1<br>AM933173.1<br>CP022963.1<br>CP012347.1<br>CP068386.1<br>LK931482.1<br>CP006575.1<br>CP075028.1<br>CP075018.1<br>CP074215.1		*CP082723.1*<br>*CP030005.1*	
**TRX F1c/B1c**	None (Expected)	–	None	Internal segments of FIP and BIP primers. Not a functional PCR pair; no amplicon is expected.
**TRX F2/B1c**	None (Expected)	–	None	Control test for non-specific pairing. Primers are not oriented to produce an amplicon;

**TRX F1c/B2**	None (Expected)	–	None	Control test for non-specific pairing. Primers are not oriented to produce an amplicon;
**invA F2/B2**	Multiple Salmonella enterica serovars (genus-conserved)	157	None	Expected for genus-level detection
**invA F1c/B1c**	None	–	None	Included for completeness (Internal segments of FIP and BIP primers. Not a functional PCR pair; no amplicon is expected.)

Primer-BLAST predicts PCR-style products. For the TRX target, the F2 and B2 primers alone appear capable of generating such products in a limited number of motile genomes (e.g., Enteritidis, Typhimurium, Berta). However, successful LAMP amplification requires the complete inner primer architecture, specifically, F2 paired with F1c and B2 paired with B1c, in the correct orientation and with proper spacing [12, 2, 3].

Supporting this, mixed inner-pair tests (F2+B1c or F1c+B2) did not produce any amplicon in motile serovars, and manual mapping of representative genomes (Enteritidis CP045955.1; Typhimurium CP088136.1) revealed also inverted order or incompatible spacing between inner primer sites. Experimentally, no TRX amplification was observed in motile serovars (10 replicates/strain; NTCs negative). Therefore, predicted F2/B2 products in PCR-style models do not imply that LAMP amplification is feasible.

1. Notomi, T.; Okayama, H.; Masubuchi, H.; Yonekawa, T.; Watanabe, K.; Amino, N.; Hase, T. Loop-Mediated Isothermal Amplifi-cation of DNA. *Nucleic Acids Res.* **2000**, *28*, e63. https://doi.org/10.1093/nar/28.12.e63.
2. Notomi, T.; Mori, Y.; Tomita, N.; Kanda, H. Loop-Mediated Isothermal Amplification (LAMP): Principle, Features, and Future Prospects. *J. Microbiol.* **2015**, *53*, 1–5. https://doi.org/10.1007/s12275-015-4656-9.
3. Gadkar, V.J.; Goldfarb, D.M.; Gantt, S.; Tilley, P.A.G. Real-Time Detection and Monitoring of Loop Mediated Amplification (LAMP) Reaction Using Self-Quenching and De-Quenching Fluorogenic Probes. *Sci. Rep.* **2018**, *8*, 5548. https://doi.org/10.1038/s41598-018-23930-1.

Table S3. Cross-dimer analysis of TRX and invA primers

Gene	Primer pair	Dimer ΔG (kcal/mol)	Notes
**invA**	**F3/B3**	-6.75	Acceptable
	**F3/LF**	-6.36	Acceptable
	**F3/ FIP**	-5.02	Acceptable
	**F3/BIP**	-8.09	Acceptable
	**F3/LB**	-4.95	Acceptable
	**B3/LF**	-3.61	Acceptable
	**B3/FIP**	-5.23	Acceptable
	**B3/BIP**	-8.02	Acceptable
	**B3/LB**	-4.95	Acceptable
	**FIP/BIP**	-11.55	Internal overlap, no 3′ complementarity
	**FIP/LF**	-6.37	Acceptable
	**FIP/LB**	-6.37	Acceptable
	**BIP/LF**	-9.98	Acceptable
	**BIP/LB**	-8.13	Acceptable
	**LF/LB**	-6.46	Acceptable
**TRX**	**F3/B3**	-5.99	Acceptable
	**F3/LF**	-3.9	Acceptable
	**F3/ FIP**	-5.99	Acceptable
	**F3/BIP**	-8.2	Acceptable
	**F3/LB**	-4.67	Acceptable
	**B3/LF**	-4.95	Acceptable
	**B3/FIP**	-12.89	Internal overlap, no 3′ complementarity
	**B3/BIP**	-6.68	Acceptable
	**B3/LB**	-7.71	Acceptable
	**FIP/BIP**	-6.96	Acceptable
	**FIP/LF**	-6.96	Acceptable
	**FIP/LB**	-5	Acceptable
	**BIP/LF**	-5.24	Acceptable
	**BIP/LB**	-8.61	Acceptable
	**LF/LB**	-6.75	Acceptable

Table S4. Ct values obtained in real-time PCR assays for invA and TRX genes across serial dilutions of Salmonella culture DNA. Four independent replicates (n = 4) were performed per dilution. Mean Ct and standard deviation (SD) were calculated for each condition. Undet. = undetermined (no amplification detected).

Gene	Sample	Rep 1 Ct	Rep 2 Ct	Rep 3 Ct	Rep 4 Ct	Mean Ct	SD of Ct
**invA**	**2.41x10^4 CFU/µl**	20.20	20.27	20.1	20.14	20.18	0.07
	**2.41x10^3 CFU/µl**	23.49	23.53	23.44	23.46	23.48	0.04
	**2.41x10^2 CFU/µl**	26.87	26.82	26.94	26.9	26.88	0.05

	**2.41x10 CFU/µl**	30.22	30.22	30.21	30.22	30.22	0.01
	**2.41 CFU/µl**	Undet.	Undet.	Undet.	Undet.	N/A	N/A
	**0.241 CFU/µl**	Undet.	Undet.	Undet.	Undet.	N/A	N/A
	**0.0241 CFU/µl**	Undet.	Undet.	Undet.	Undet.	N/A	N/A
	**NC**	Undet.	Undet.	Undet.	Undet.	N/A	N/A
**TRX**	**1.65x10^5 CFU/µl**	20.16	20.21	20.12	20.14	20.16	0.04
	**1.65x10^4 CFU/µl**	23.63	23.61	23.65	23.63	23.63	0.02
	**1.65x10^3 CFU/µl**	27.05	27.09	27.02	27.06	27.06	0.03
	**1.65x10^2 CFU/µl**	30.56	30.54	30.59	30.58	30.57	0.02
	**1.65x10 CFU/µl**	33.88	33.91	33.85	33.86	33.88	0.03
	**1.65 CFU/µl**	Undet.	Undet.	Undet.	Undet.	N/A	N/A
	**0.165 CFU/µl**	Undet.	Undet.	Undet.	Undet.	N/A	N/A
	**NC**	Undet.	Undet.	Undet.	Undet.	N/A	N/A
